# Supplementary material for: Magnetic slippery extreme icephobic surfaces
Source: Nat Commun. 2016 Nov 8;7:13395. doi: 10.1038/ncomms13395 (PMC5105164; doi:10.1038/ncomms13395)
Supplement: Supplementary Information — Supplementary Figures 1-21, Supplementary Tables 1-2, Supplementary Notes 1-7 and Supplementary References [file ncomms13395-s1.pdf]

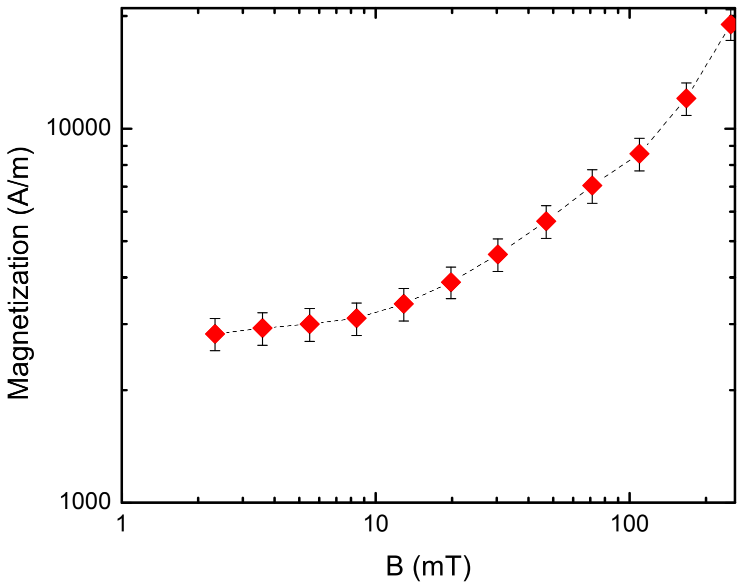

Supplementary Figure 1: Magnetization of the studied ferrofluid is measured as a function of the imposed magnetic field. We used the Gouy method in these experiments. Choice of this ferrofluid was based on its high saturation magnetization.

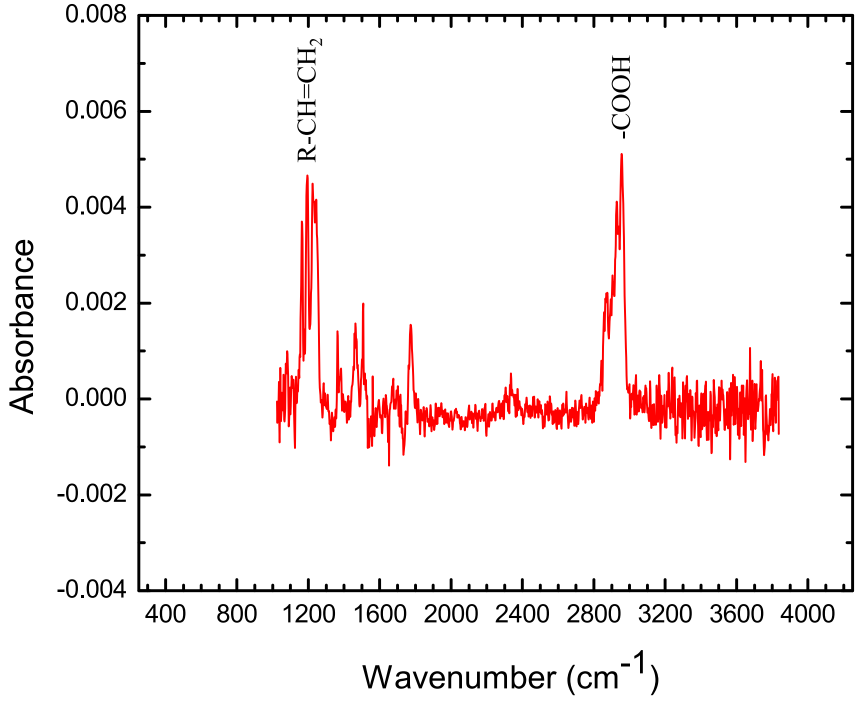

Supplementary Figure 2: The chemical structure of ferrofluid studied in this work was analyzed by FTIR. The ferrofluid mostly contain fatty acids, primarily Lauric acic ($CH_3(CH_2)_{10}COOH$).

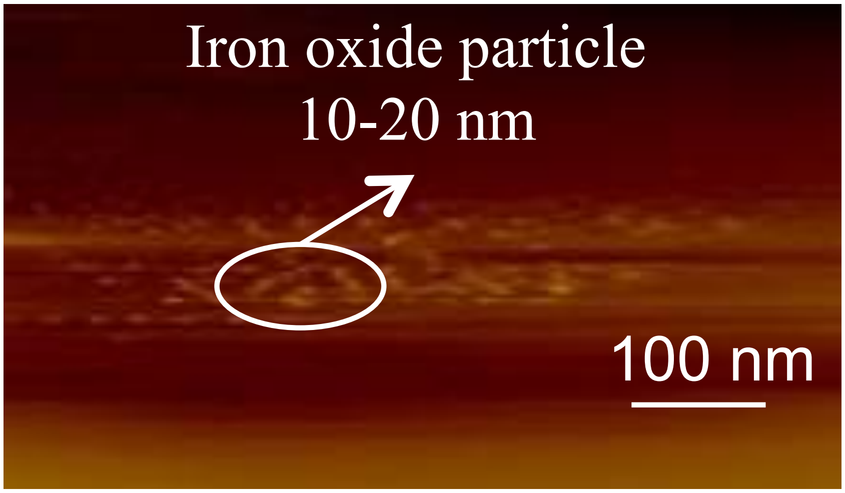

Supplementary Figure 3: The dimension of iron-oxide particles in the ferrofluid was examined by Scanning Probe Microscopy (SPM). For a diluted droplet of ferrofluid, the coffee-ring effect leaves traces of particles on a smooth Si wafer. The trace includes clusters of iron oxide particles. The smallest particles found in the trace are in the range of 10-20 nm.

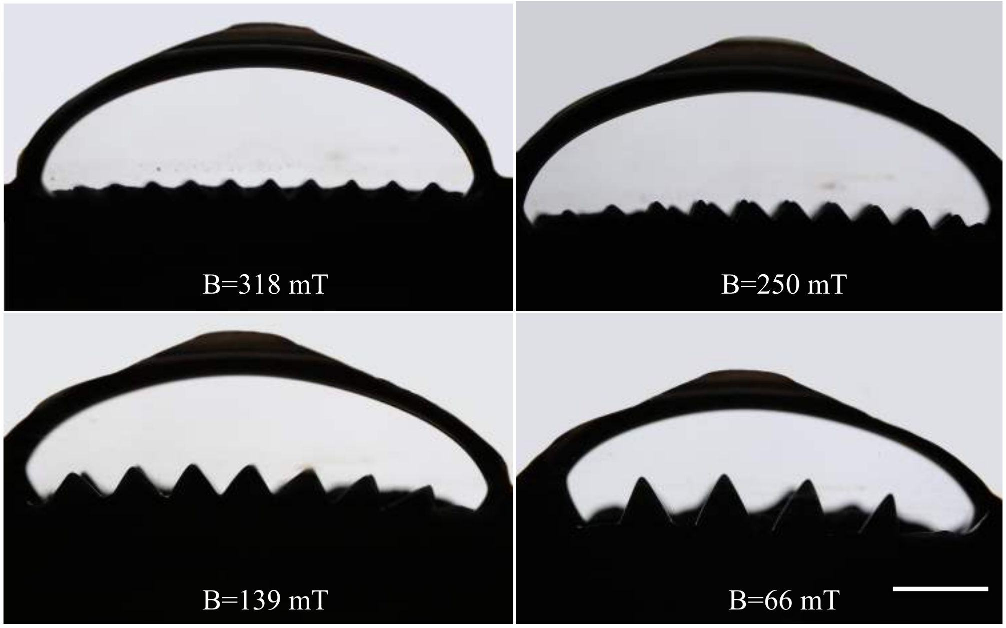

Supplementary Figure 4: The introduced surface waves as a function of the magnetic field in water-ferrofluid system are shown. The amplitude of the waves is a decreasing function of the magnetic field. The scale bar in the pictures represents 1 mm.

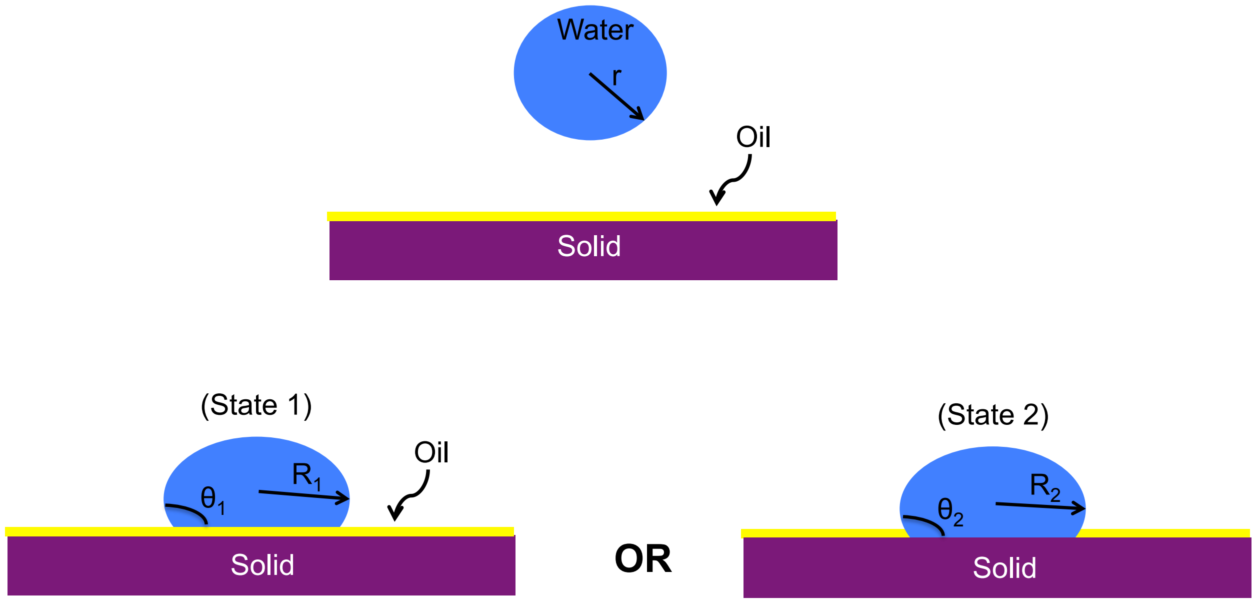

Supplementary Figure 5: Once a water droplet sits on SLIPS, two configurations can occur at the solid interface. In state 1, a solid-oil interface and a water-oil interface coexist. In state 2, these two interfaces are replaced by a solid-water interface.

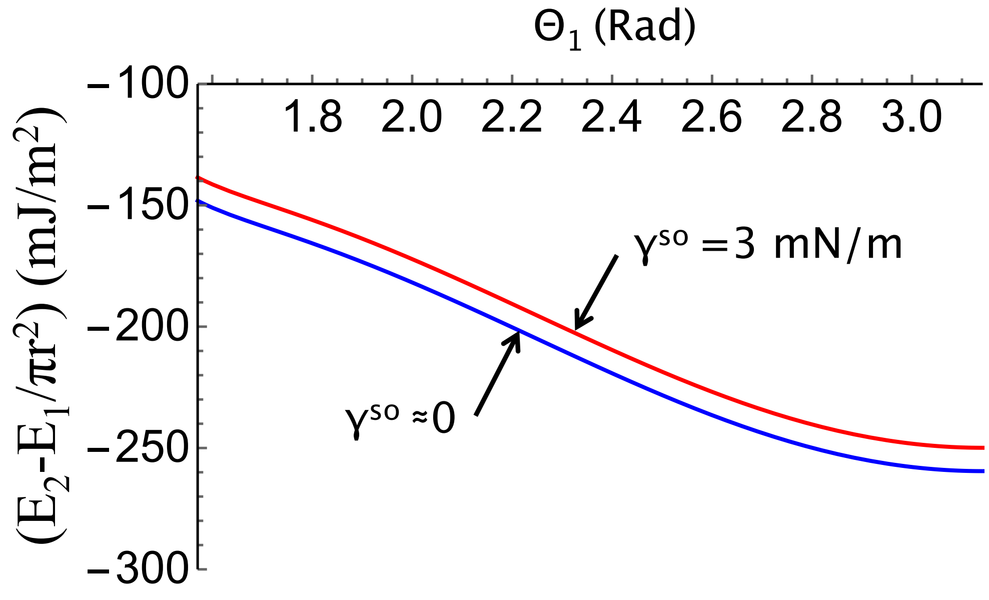

Supplementary Figure 6: The differential energy of state 1 and state 2 are calculated. The findings suggest that state 2 is the stable thermodynamic state and a solid-water interface can form for a droplet sitting on SLIPS.

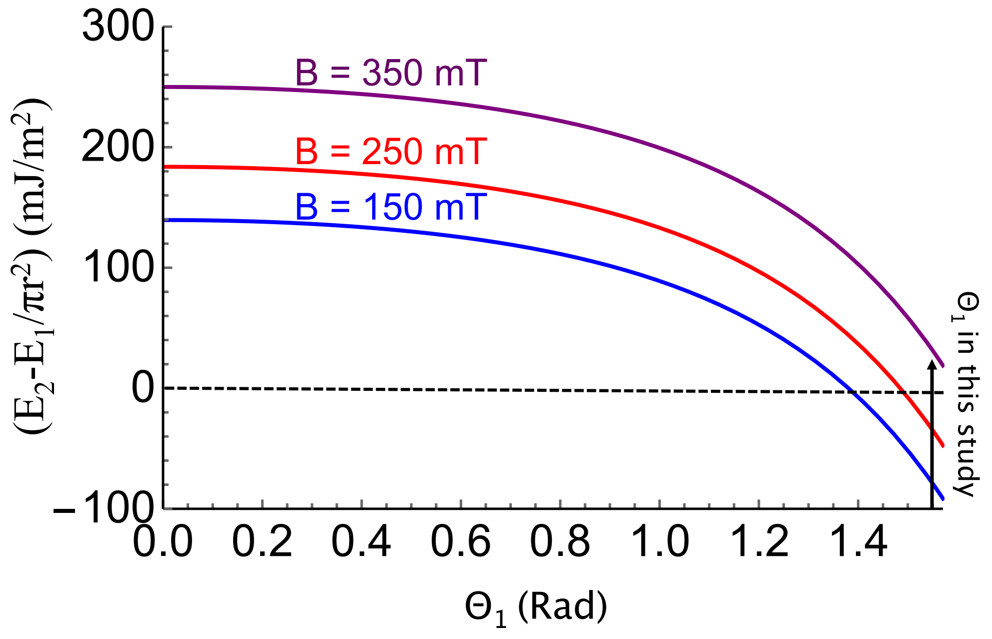

Supplementary Figure 7: The differential energy of state 1 and state 2 is calculated by including the volumetric magnetic energy. The results suggest that state 1 is stable at high magnetic field and the solid-water interface is completely excluded.

Experimental Setup

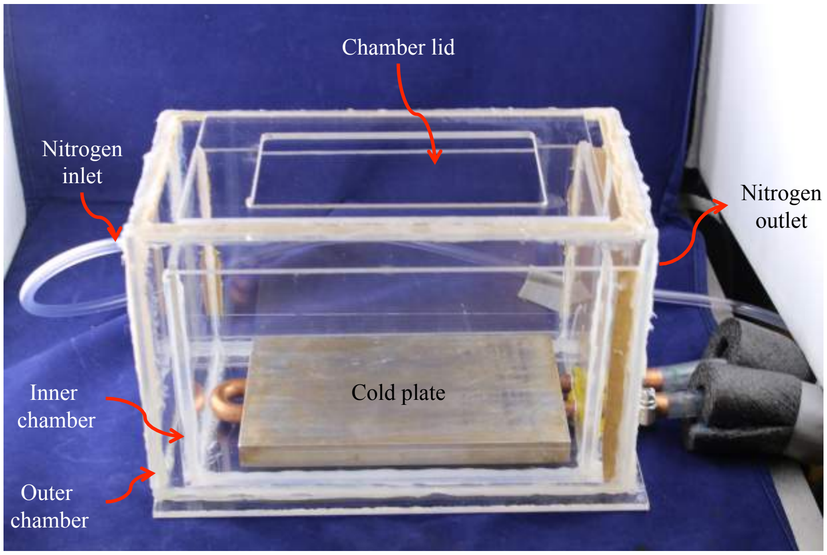

Supplementary Figure 8: The experimental chamber for ice nucleation measurements is shown. Cold nitrogen gas flows over the aluminum cold plate inside the chamber. Refrigerant enters and exits on the right-hand side. Nitrogen enters on the left and exits on the right. Note that the inner chamber is introduced to avoid the role of nitrogen flow on the ice formation. The inner chamber is open-ended on top.

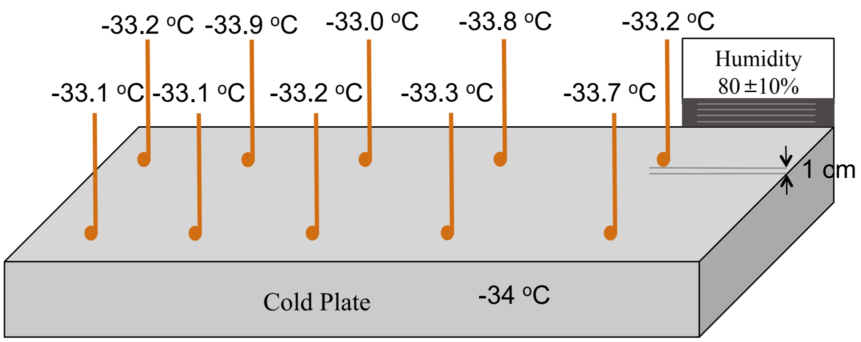

Supplementary Figure 9: The temperature in the icing test chamber was measured at 10 points above the cold plate. The temperature variation in the chamber was ± 1°C and the humidity in the chamber was 80 ±10 %

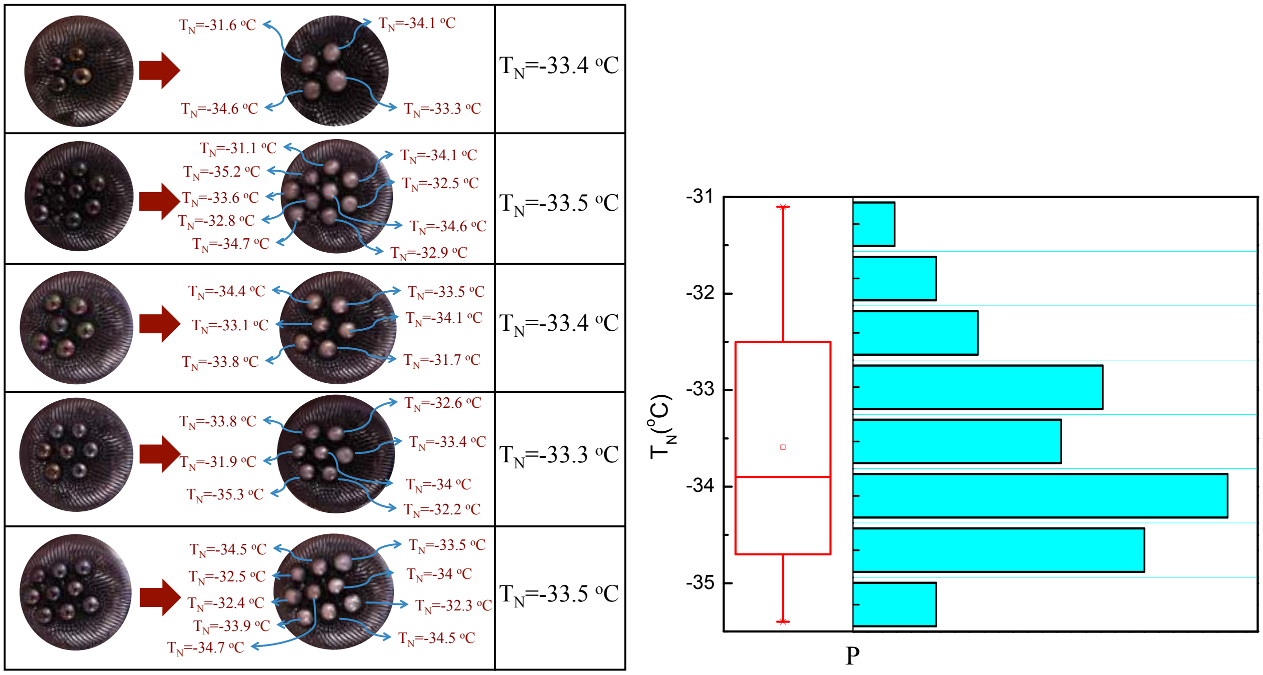

Supplementary Figure 10: (a) Pictures of the conducted experiments to find T_N are shown. (b) The median and the error bar on the T_N measurements are shown. Also, the probability density of measured data is represented on the right-hand-side sub-figure.

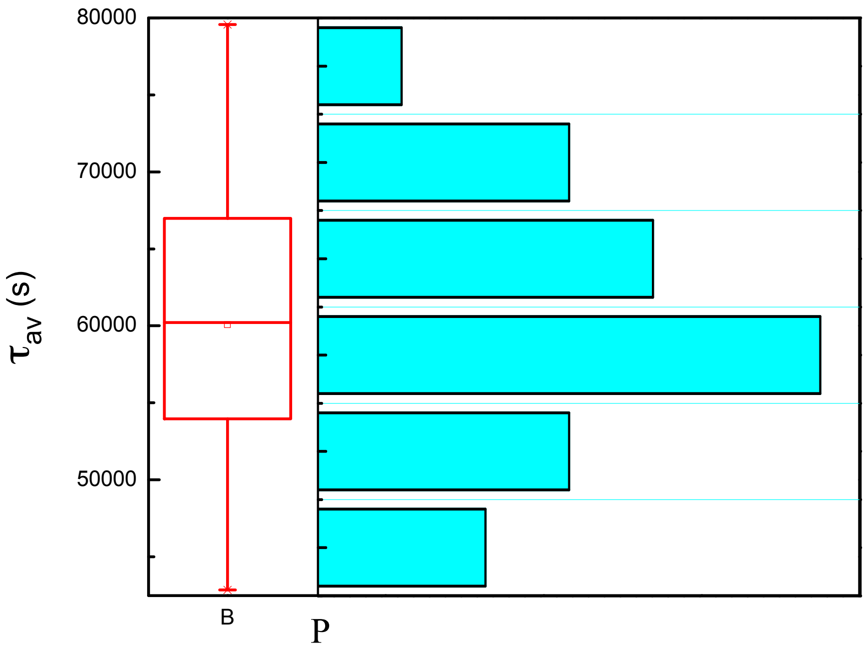

Supplementary Figure 11: The measured ice nucleation delay time at a temperature of -25 °C on MAGSS is shown. In addition, the probability density of the 19 measurements is shown.

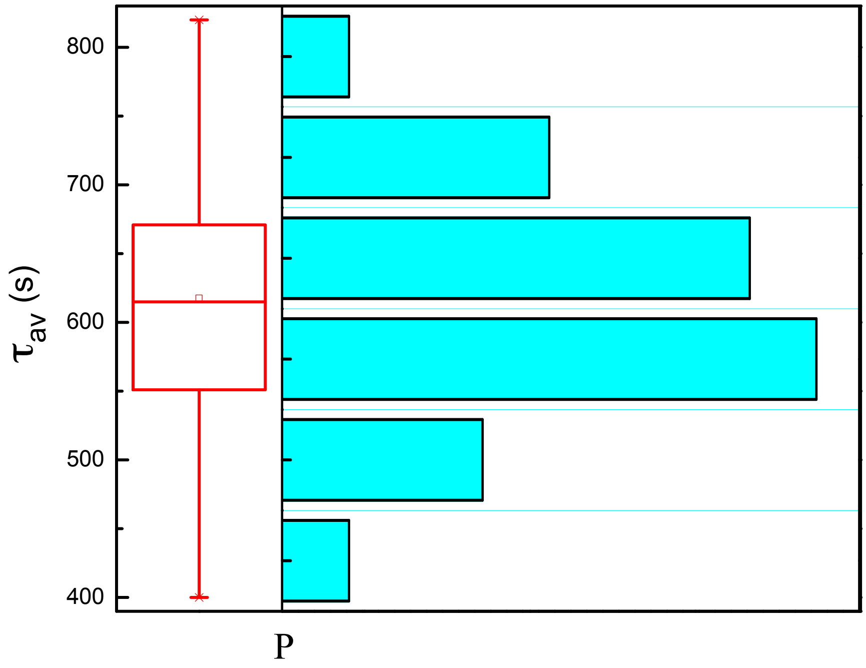

Supplementary Figure 12: The measured ice nucleation delay time at a temperature of -30 °C on MAGSS is shown. In addition, the probability density of the 24 measurements is shown.

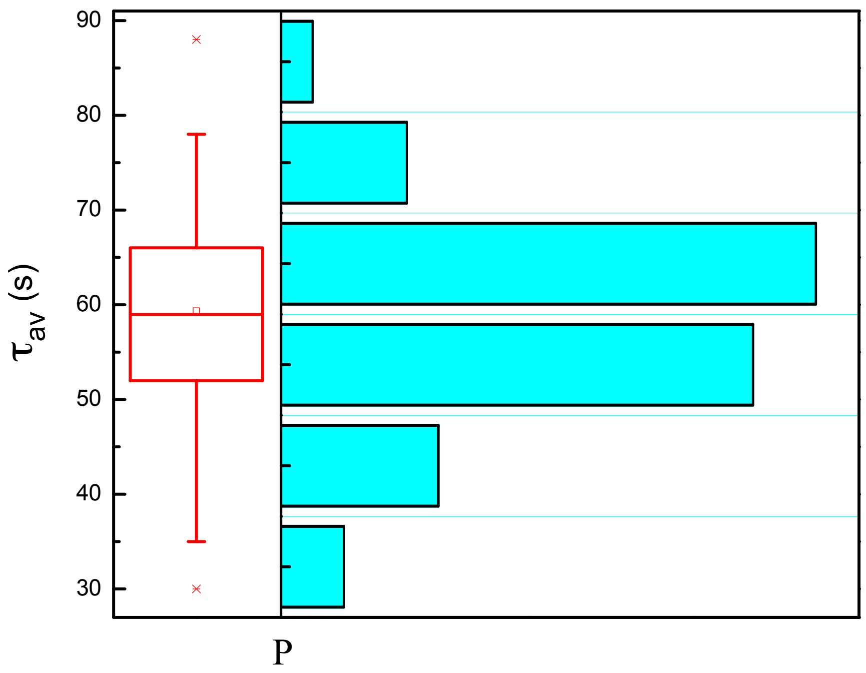

Supplementary Figure 13: The measured ice nucleation delay time at a temperature of -34 °C on MAGSS is shown. In addition, the probability density of the 44 measurements is shown.

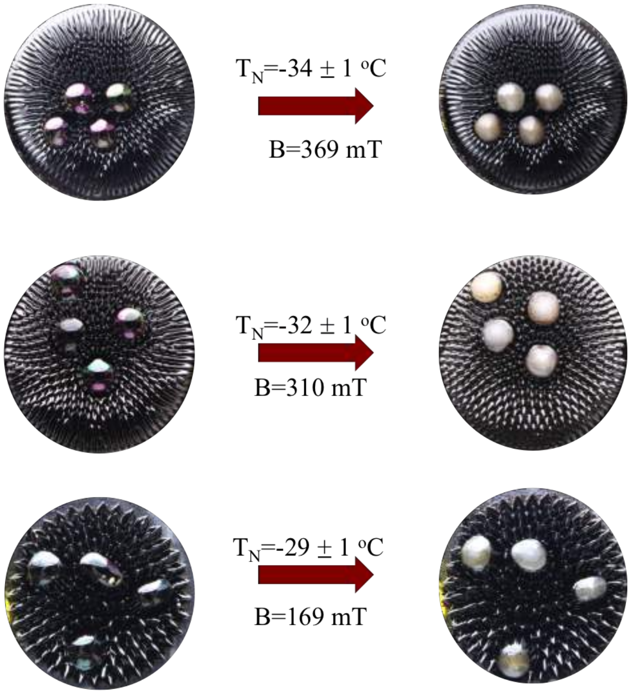

Supplementary Figure 14: The ice formation on MAGSS at three magnetic fields is shown. At low magnetic fields, the partial existence of a solid-water interface increases the ice formation temperature. However, at high magnetic fields, the solid-water interface is completely excluded and a T_N value of -34 $\pm$ 1 °C is achieved. With further increase in the magnetic field, we did not observe any change in the ice nucleation temperature and the ice nucleation delay time.

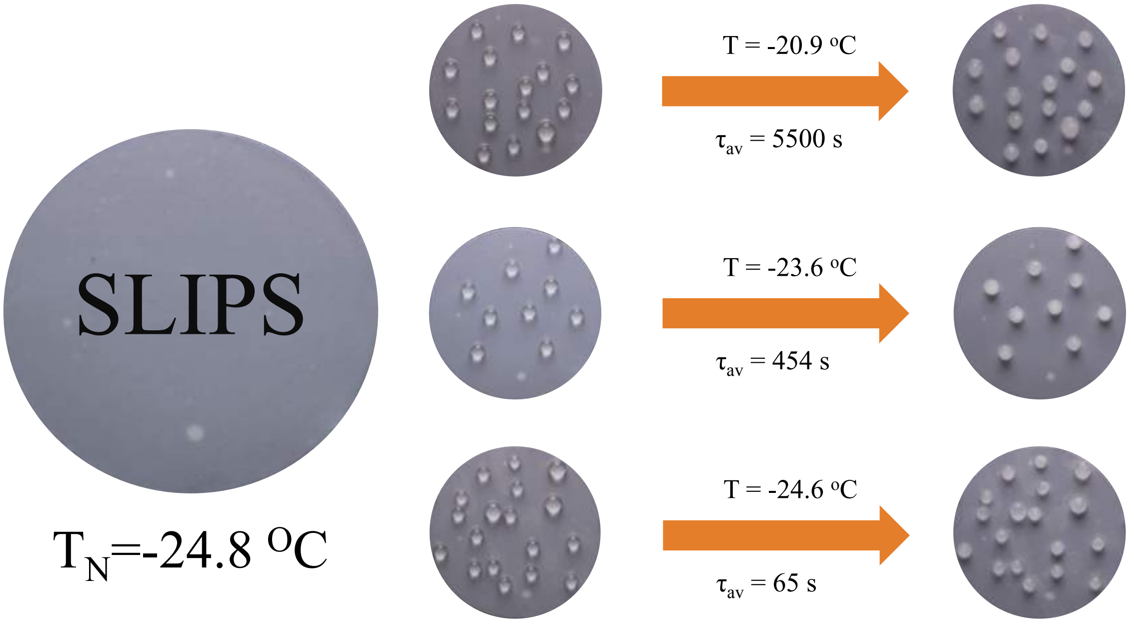

Supplementary Figure 15: Icephobicity of SLIPS was examined in the icing test chamber. The measured value of T_N for SLIPS is -25 ± 1 °C, similar to the previously reported values.[1] (b) We also measured the average ice nucleation delay time on SLIPS as a function of temperature.

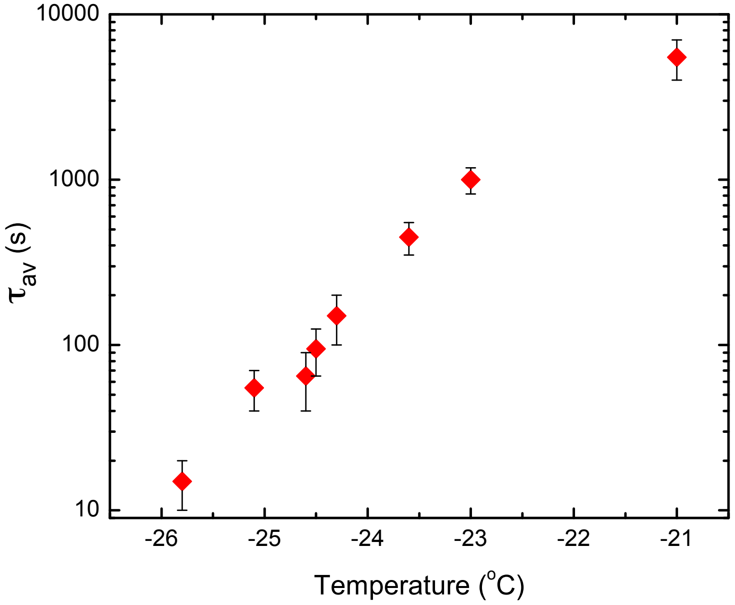

Supplementary Figure 16: The average ice nucleation delay time on SLIPS as a function of temperature is measured in the icing test chamber.

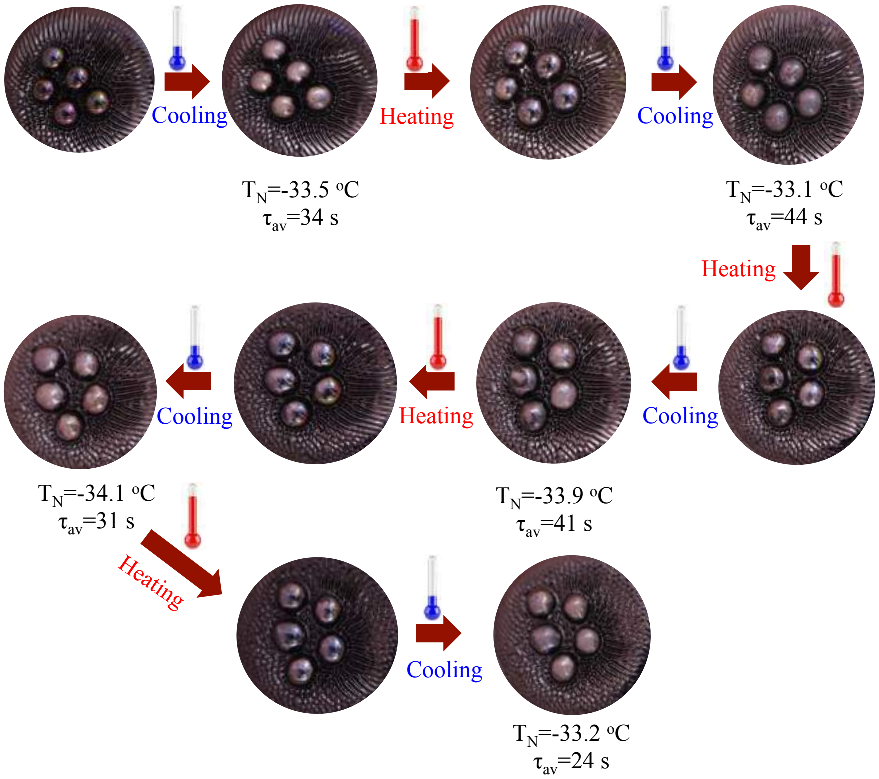

Supplementary Figure 17: The role of heating/cooling cycles on icephobicity of MAGSS is studied. The DI water droplets are deposited on a MAGSS surface and are subjected to five heating/cooling cycle. The change in T_N is $\pm$ 0.5°and the change in τ_{av} is $\pm$ 10s. No change in icephobicity of MAGSS was observed.

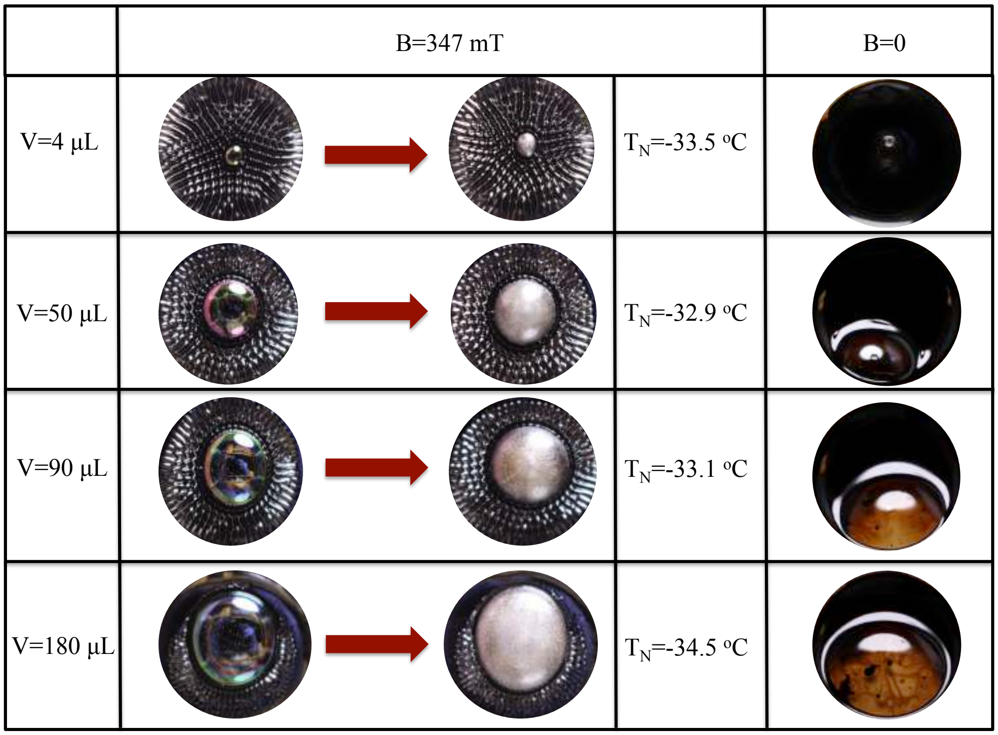

Supplementary Figure 18: Role of the droplet volume on the icephobicity of MAGSS was studied. We could not detect any measurable difference in the icephobicity of MAGSS as a function of droplet volume in the studied range. Also, on the right-hand side, we show that in the absence of a magnetic field, the droplet sinks in the ferrofluid layer.

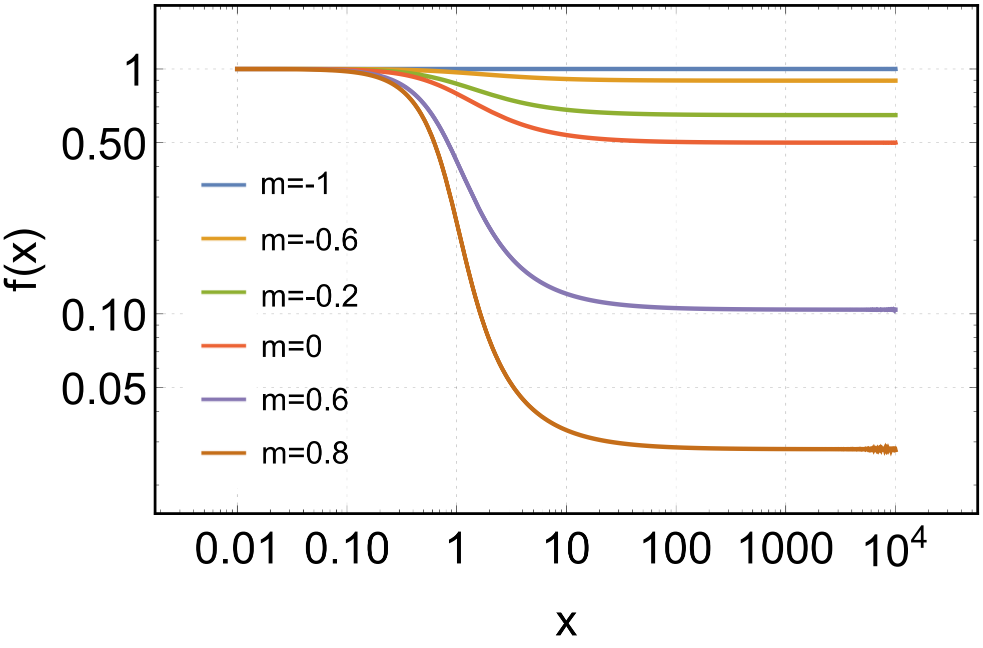

Supplementary Figure 19: The geometrical factor of heterogenous ice nucleation is plotted as a function of x for a range of values of m. At high values of x, f (m,x) becomes independent of x and is only a function of m.

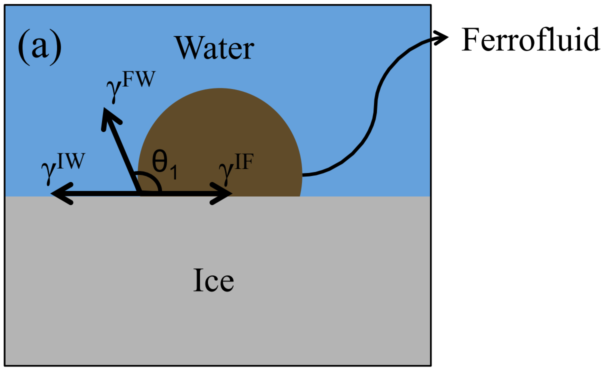

Supplementary Figure 20: Two cases are considered to determine the value of m in MAGSS. (a) Ferrofluid droplet on ice substrate in water environment and (b) ice embryo on ferrofluid in water environment.

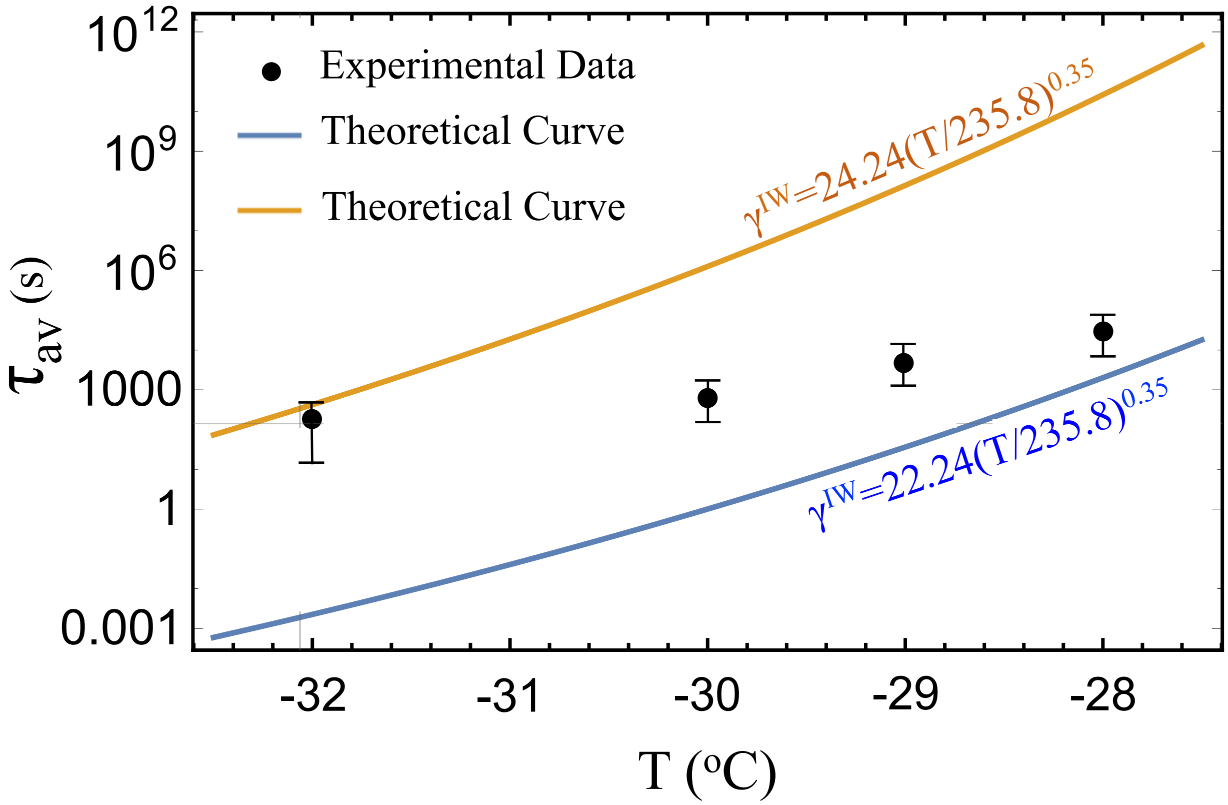

Supplementary Figure 21: Through measured values of m, we predicted the ice nucleation delay time through the nucleation theory and compared with the measured values. The measured values are within the uncertainty of the predictions.

Supplementary Table 1: Measured values of surface tension of ferrofluid-air and ferrofluid-water interfaces

T (°C)	B (mT)	γ^F(mNm^{-1})	γ^{FW}(mNm^{-1})
4	0	-	18.5
10.5	0	-	19.5
22	0	24.1	22.8
22	43	24.1	22.8
22	70	24.1	22.8
22	112	24.1	22.8
22	318	24.1	22.8

Supplementary Table 2: Measured ice nucleation delay time on MAGSS as a function of magnetic field

	-25 ^oC		-30 ^oC		-34 ^oC	
B	t (s)	Frozen drop.	t (s)	Frozen drop.	t (s)	Frozen drop.
37 mT	38-44	2	9-12	3	0.8-1.3	1
	44-50	5	12-15	4	1.3-1.8	3
	50-56	6	15-18	7	1.8-2.3	7
	56-62	2	18-21	1	2.3-2.8	4
42 mT	44-52	1	12-16	1	2.5-3.5	2
	52-60	5	16-20	3	3.5-4.5	3
	60-68	7	20-24	7	20-24	7
	68-76	2	24-28	4	5.5-6.5	2
165 mT	350-400	1	73-93	3	5-7	1
	400-450	4	93-113	4	7-9	9
	450-500	9	113-133	6	9-11	3
	500-550	1	133-153	2	11-15	2
310 mT	5934-6684	2	210-255	1	15-21	1
	6684-7434	4	255-300	3	21-27	6
	7434-8184	8	300-345	8	27-33	7
	8184-8934	1	345-390	3	33-39	1
347 mT	24000-27000	3	250-325	2	26-34	2
	27000-30000	5	325-400	2	34-42	6
	30000-33000	5	400-475	6	42-50	6
	33000-36000	2	475-550	5	50-58	1

Supplementary Note 1- Surface tension measurements

Surface tension of the ferrofluid was measured with a tensiometer (Biolin Scientific, Sigma 701) equipped with a Du Nuoy ring. Ferrofluid-water interfacial tension at different magnetic fields and temperatures were measured in the same manner. The results of these experiments are tabulated in Table S1.

Supplementary Note 2- Surface waves at the ferrofluid-water interface

Once a magnetic fluid is exposed to a magnetic field, surface waves can appear depending on the involved opposing forces. In Rosensweig instability[2], once the ferrofluid is exposed to a homogenous magnetic field, surface waves appear and the critical wavelength at the onset of instability is given by

$$\lambda_c = 2\pi\sqrt{\frac{\gamma}{\rho g}} \tag{1}$$

where γ denotes surface tension of ferrofluid interface, ρ density of the ferrofluid, and g gravitational acceleration.This value is in the range 1 cm for ferrofluid-water interface. Once the gravitational field becomes negligible compared to the magnetic field, the wavelength of the surface waves is determined by the competition between surface tension force and the volumetric magnetic force. In this regime, only one recent study by Timonen et al.[3] suggested an approximate criteria for the observed wavelengths. These authors studied the dynamic self-assembly of ferrofluid droplets on a superhydrophobic surface. They found that the wavelength for splitting of a ferrofluid droplet to

several droplets is given approximately by

$$\lambda_c \approx 2\pi \sqrt{\frac{\gamma}{\frac{d}{dz}(\mu_0 HM)}} \tag{2}$$

where μ_0 denotes permeability of vacuum, H field strength, and M magnetization of the ferrofluid. This wavelength suggests a criteria for splitting of a ferrofluid droplet and *not* wavelength of continuous waves on the ferrofluid surface. None of these wavelength criteria can be applied directly to our work. Based on our study, we suggest that the observed surface waves are a competition between the surface tension force and the magnetic volumetric force. As we increase magnetic volumetric force (or reduce surface tension of ferrofluid), the wavelength and amplitude of these waves can be reduced. In Fig. 4, the change in the amplitude of the surface waves as a function of the induced magnetic field is shown.

Supplementary Note 3- Thermodynamic states of a water droplet on MAGSS

Here, we present a thermodynamic analysis to determine the stable state of a water droplet on SLIPS first and after on MAGSS. Once a water droplet sits on SLIPS, two thermodynamic states can occur at the solid surface as shown in Fig. 5. In state 1, a solid-oil interface and a water-oil interface coexist. In state 2, these two interfaces are replaced by a solid-water interface. Here, the radius of studied droplet is smaller than the capillary length and consequently the role of gravity in this analysis is neglected.

Initially, we should study the constraint on volume of the water droplet. The initial droplet has a radius of r. The siting droplet in state 1, has a radius of R_1 and a contact angle of θ_1. Thus,

according to Young equation,

$$\cos\theta_1 = \frac{\gamma^{oa} - \gamma^{ow}}{\gamma^{wa}} \tag{3}$$

where γ denotes the surface tension, superscript o oil, superscript a air, superscript w water, and superscript s solid. For SLIPS, γ^{oa} is 17 mN/m and γ^{ow} is 56 mN/m [4]. Thus, the numerator is a negative value and $\theta_1 \geq \pi/2$. Thus, the constraint on the volume of a water droplet in state 1 results in

$$R_1 = b_1^{1/3} r$$
$$b_1 = [1 - \frac{(1+\cos\theta_1)^2(2+\cos\theta_1)}{4}]^{-1} \tag{4}$$

For state 2, the Young equation is written as

$$\cos\theta_2 = \frac{\gamma^{so} - \gamma^{sw}}{\gamma^{ow}} \tag{5}$$

For SLIPS, the spreading parameter, which is written as $S = \gamma^s - \gamma^{oa} - \gamma^{so}$ should be positive. As γ^s is 20 mN/m, and γ^{oa} is 17 mN/m, γ^{so} should be *less* than 3 mN/m. Lets consider a water droplet siting on the solid surface. The Young equation for this droplet is written as $\gamma^s - \gamma^{sw} = \gamma^{wa}\cos\theta_w$. As γ^s is 20 mN/m, γ^{wa} is 72 mN/m, and θ_w is 113° for SLIPS [4], one finds that γ^{sw}= 48.13 mN/m. Looking back at Eq. 5, the numerator is negative and the volumetric constraint for state 2 leads to

$$R_2 = b_2^{1/3} r$$
$$b_2 = [1 - \frac{(1+\cos\theta_2)^2(2+\cos\theta_2)}{4}]^{-1} \tag{6}$$

The interfacial energies in the state 1 and state 2 are written as

$$E_1 = \pi R_1^2 \gamma^{so} + \pi R_1^2 \sin^2\theta_1 \gamma^{ow} + \pi R_1^2 (1 - \sin^2\theta_1)\gamma^{oa} + \gamma^{wa} A_1$$
$$E_2 = (\pi R_2^2 \sin^2\theta_2)\gamma^{sw} + (\pi R_1^2 - \pi R_2^2 \sin^2\theta_2)(\gamma^{so} + \gamma^{oa}) + \gamma^{wa} A_2 \tag{7}$$

Where A_1 and A_2 represent the surface area of the water-air interface and are written as

$$A_1 = 2\pi R_1^2(1 + \cos\theta_1)$$
$$A_1 = 2\pi r^2 b_1^{2/3}(1 + \cos\theta_1) \quad (8)$$

And

$$A_2 = 2\pi R_2^2(1 + \cos\theta_2)$$
$$A_2 = 2\pi r^2 b_2^{2/3}(1 + \cos\theta_2) \quad (9)$$

Note that if oil cloaks the droplet, the γ^{wa} in the last terms of Eqs. 7 is replaced by $(\gamma^{ow} + \gamma^{oa})$. Thus, the differential of energy between state 1 and state 2 is written as

$$E_2 - E_1 = (\gamma^{sw} - \gamma^{so} - \gamma^{oa})(\pi R_2^2 \sin^2\theta_2) +$$
$$(\gamma^{oa} - \gamma^{ow})(\pi R_1^2 \sin^2\theta_1) + \gamma^{wa}(A_2 - A_1) \quad (10)$$

or by using Eqs. 3 and 5,

$$\frac{(E_2 - E_1)}{\pi r^2} = (\gamma^{sw} - \gamma^{so} - \gamma^{oa})(b_2^{2/3}\sin^2\theta_2) +$$
$$(\gamma^{oa} - \gamma^{ow})(b_1^{2/3}\sin^2\theta_1) + 2\gamma^{wa}(b_2^{2/3}(1 + \cos\theta_2) - b_1^{2/3}(1 + \cos\theta_1)) \quad (11)$$

Note that the final differential energy equation is only a function of interfacial tensions. For SLIPS, we considered two extreme cases: (I) γ^{so} approaches zero, in which θ_2 is 143° and (II) γ^{so} approaches 3 mN/m in which θ_2 is 149°. For both of these cases, we plotted the differential energy as a function of θ_1 in Fig. 6. The results suggest that state 2 is the stable thermodynamic state and a solid-water interface forms in SLIPS. We emphasize that in state 1, there are two interfaces

involved, water-oil and solid-oil, while in state 2 these interfaces are replaced by one interface of a solid-water interface.

Now, we modify the energy equation by including the magnetic volumetric force. For the ferrofluid studied here, the contact angle of water on the ferrofluid surface θ_1 is *less* than π/2. Thus, the form of equation for b_1 is written as

$$R_1 = b_1^{1/3} r$$
$$b_1 = [\frac{(1-\cos\theta_1)^2(2+\cos\theta_1)}{4}]^{-1} \tag{12}$$

By including the volumetric magnetic energy in Eq. 11, the differential energy equation is written as

$$\frac{(E_2 - E_1)}{\pi r^2} = (\gamma^{sw} - \gamma^{so} - \gamma^{oa})(b_2^{2/3}\sin^2\theta_2) +$$
$$(\gamma^{oa} - \gamma^{ow})(b_1^{2/3}\sin^2\theta_1) + 2\gamma^{wa}(b_2^{2/3}(1+\cos\theta_2) - b_1^{2/3}(1+\cos\theta_1)) + \frac{1}{2}\mathbf{f}_m t^2 \tag{13}$$

where $\mathbf{f}_m$ denotes the magnetic body force density and t thickness of the ferrofluid layer (300 μm). The magnetic body force is equal to $\mu_0\chi\mathbf{H}^2$, where μ_0 denotes the vacuum magnetic permeability, $\chi = \frac{M}{H}$ is the susceptibility of the ferrofluid, and $\mathbf{H}$ is the magnetic strength. The magnetic strength is expressed as $\mathbf{H} = \mathbf{B}\mu^{-1}(1+\chi)^{-1}$, where $\mathbf{B}$ denotes induction magnetic field. The modified differential equation as a function of θ_1 is shown in Fig. 7 as a function of the applied magnetic field.

As shown, the magnetic field in the order of 300 mT is required to *completely* exclude the solid-water interface. Any partial existence of this interface leads to ice nucleation at high temperatures. We should emphasize that in these calculations, the solid plays a role in γ^{sw} and γ^{so}. These

calculations can be conducted for any combination of oil and solid once the surface tension values are available.

Furthermore, we show the exclusion of the solid-water interface in MAGSS in supplementary Movie 1. A Si wafer is obtained and cleaned with a Plasma cleaner. A layer of ferrofluid is deposited on the Si wafer. The thermodynamic equilibrium requires formation of a solid-water interface as can be seen clearly in the video (Supplementary Movie 1) the center of the droplet turn to a bright color. Formation of a solid-water interface leads to pinning of the droplet as shown in the Movie. Then, a permanent magnet is placed under the Si substrate to induce the volumetric magnetic force. The solid-water interface disappers and ferrofluid-water interface is formed. The high mobility of the droplet on MAGSS is an indication of the formation of the ferrofluid-water interface.

In SLIPS, the surface is a combination of (I) flat solid areas (between the pores) and (II) pores filled with oil. Once a water droplet sits on SLIPS, thermodynamic equilibrium requires the formation of a solid-water interface on the flat area. To show the formation of this solid-water interface, we conducted an experiment shown in Supplementary Movie 3. In this video, we compare the mobility of a droplet on a SLIPS surface and MAGSS. We emphasize that the viscosity of FC-70 and ferrofluid herein are close (FC-70, 24 mPas and ferrofluid, 6 mPas). If in both cases, only the water-liquid interface is present, a water droplet should have the same mobility on these surfaces. However, any *partial* formation of a solid-water interface will reduce the mobility of the droplet. As shown in supplementary Movie 3, the water mobility on MAGSS is much higher than that of SLIPS, suggesting that the partial formation of a solid-water interface occurs in SLIPS.

Supplementary Note 4- Experimental setup

All T_N and τ_{av} experiments were conducted in an icing test chamber assembled from acrylic plates. The aluminum cold plate was inserted into the chamber and each MAGSS sample was placed on the cold plate. The refrigerant from the chiller cooled the cold plate to a desired temperature. Before the start of each experiment, the chamber was purged of ambient air by flowing nitrogen through the chamber for 2-3 minutes. The nitrogen gas tube was placed inside the chiller and the nitrogen gas was allowed to reach the same temperature as the cold plate before flowing through the chamber. The chamber for the ice formation studies is shown in Fig. 8. The temperature in the icing test chamber was measured at 10 points. The temperature fluctuation in the test chamber was ± 1 °C. The humidity of the test chamber was controlled with a humidity sensor (Traceable, thermo-hygro). This humidity was maintained at 80 ± 10 % in the experiments (see Fig. 9).

Supplementary Note 5- Experimental procedures

Median ice nucleation temperature (T_N) The median nucleation temperatures were measured for MAGSS with different applied magnetic fields. The sample was placed in the nitrogen chamber with a starting temperature of -20°C and cooling rate of 1°C/10 min. A droplet of distilled water (volume of 30μL) was added at the starting temperature. Two thermocouples were installed to measure temperature of the sample near the droplet. The thermocouples were attached to the sample with thermal paste (Omega, Omegatherm 201). The temperature of the sample was recorded

as close as possible to the droplet without causing disturbance to the experiment. The median ice nucleation temperature was measured for 35 droplets as shown in Fig. 10(a). Furthermore, the box plots for the measured T_N and probability density of the measurements are shown in Fig. 10(b).

Average nucleation delay time (τ_{av}) The nucleation delay time, or time for nucleation to occur, was measured for MAGSS at different temperatures and with different applied magnetic fields. The water droplet was placed at the center of the sample in each experiment. For all of the experiments, we captured the freezing behavior with a digital camera viewing from the top of the chamber. Due to the limitations of our nitrogen gas tank, we could not run any individual experiment for more than 27 hours. The ice nucleation delay time for all experiment conducted are shown below: 44 experiments for temperature of -34 °C ; 24 experiments for temperature of -30 °C ; and 19 experiments for temperature of -25 °C . In all of the measurements, the magnetic field was B=490 mT. We show the median and probability density of the average nucleation delay time in Figs. 11-13. In the presence of a magnetic field, the competition between the surface tension force and the magnetic force results into the formation of surface waves with a specific periodicity. The water residing in the pits of these surface waves can be in contact with the solid-substrate depending on the magnetic field. This partial existence of solid-water interface promotes heterogenous ice nucleation as seen for B $\leq$ 347 mT. However, once the magnetic field reaches a threshold value, as shown mathematically in Fig. 7, the solid-water interface is excluded and the ice nucleation time remains approximately constant. With further increase in the magnetic field, we did not observe any change in the ice nucleation temperature and ice nucleation delay time as seen in Fig. 14. We measured the average ice nucleation time at temperatures of -25, -30 and -34 °C at different mag-

netic fields. All these measurements are tabulated in Table 2. The box plot of these measurements are shown in Fig. 2 of the manuscript.

For comparison, the T_N value for SLIPS is examined in our icing test chamber. We developed SLIPS with a PFTE membrane (Sterlitech Corporation) and FC-70 oil (Sigma-Aldrich) exactly similar to the procedure outlined in Wong et al.[4]. We conducted the icephobicity measurement on SLIPS in our icing test chamber in the same experimental conditions as shown in Figs. 15 and 16. The measured value of T_N for SLIPS is -25 $\pm$ 1 ^{o}C.

Icephobicity in heating/cooling cycles The role of heating/cooling cycle on the measured values of T_N and τ_{av} is studied. In these cyclic experiments, the MAGSS was placed in the icing test chamber and water droplets were deposited on the MAGSS. The temperature of the MAGSS in a quasi-steady approach is reduced and T_N and τ_{av} are measured. Once all of the droplets were frozen, the MAGSS was heated to 5 °C to form water droplets again. The MAGSS was cooled again to lower temperatures and T_N and τ_{av} were measured. We conducted five cycles of heating and cooling as shown in Fig. 17. The change in T_N is $\pm$ 0.5°and the change in τ_{av} is $\pm$ 10s. The extreme icephobicity of MAGSS is unaffected by heating/cooling cycles.

Ice adhesion As ice adhesion strength on MAGSS is so small, the induced gravitational force by titling was used to determine ice adhesion strength on MAGSS. We placed MAGSS in the ice test chamber and deposited a water droplet on the surface. The temperature of the test chamber was reduced to -34 °C to form ice. Next, the test chamber was tilted stepwise with 1° tilt in each step. The threshold tilt angle for slide of ice on MAGSS was measured. Through measured threshold

tilt angle, mass of ice and the contact area of ice, we determined the adhesion strength of ice on MAGSS.

Droplet mobility Experiments were conducted with both water droplets and ice to study the extent of pinning of each on MAGSS. These experiments were conducted with magnetic tape (McMaster) with magnetic fields ranging from 20 to 52 mT. We examined droplet mobility on MAGSS at a range of droplet dimensions. For ice mobility on MAGSS, experiments were conducted with different substrates (Silicon, Glass, Acrylic) to show that the performance of MAGSS is independent of the underlying substrate. The entire ice test chamber was tilted to an angle of 2.5 ° for mobility measurements. The motion of droplet and ice was captured at a rate of 4 fps with a Canon DSLR in continuous shooting mode. The mobility of ice and water on MAGSS is shown in Supplementary Movies 2-5, respectively.

Droplet impact The self-healing characteristics of MAGSS were assessed by dropping water droplets with a range of Weber numbers on MAGSS. The magnetic field was 165 mT. The droplet size was 30 μL and was released from a syringe using a syringe pump (kdScientific). The impact was captured using a high-speed camera (Phantom V711) and a macro lens. Supplementary Movie 6 and 7 show the self-healing characteristics of MAGSS. The Weber number for these experiments were in the range of 15-600. For higher Weber numbers, we used higher viscosity ferrofluid as shown in supplementary Movie 7. Furthermore, we examined the self-healing characteristics of MAGSS under scratching by using a sharp object as shown in supplementary Movie 8. The surface of MAGSS re-heals itself fast and is unaffected by the sharp object.

MAGSS stability at high shear flows To examine the stability of MAGSS under high shear flows, we developed an experimental setup consisting of an acrylic 25 mm square-shaped tube, a nitrogen tank, a flow meter, and a digital camera. MAGSS was placed inside the Acrylic tube and a flow of nitrogen gas was introduced into the tube. The temporal behavior of MAGSS under the Nitrogen flow was examined with a digital camera. We ran these experiments for more than 15 hrs to detect any instability on MAGSS. The Movie of these experiments is included in the Supplementary Movie 9. The assumed critical threshold was depletion rate of (≤ 2 μm/hr). Furthermore, we ran the stability experiments with water as the flowing fluid. We could not detect any instability under the flow of water, even up to a Reynolds number of 2000 (Supplementary Movie 10). The water system in our lab did not allow us to conduct experiments at a higher flow rate of water.

Supplementary Note 6- Role of droplet size on median ice nucleation temperature

We studied the role of droplet dimension on the value of T_N. We could not find any measurable change in the value of T_N as a function of volume of the droplet as shown in Fig. 18. Also, in this figure, we showed that in the absence of a magnetic field, the water droplet sinks in the ferrofluid layer and forms a solid-water interface.

Supplementary Note 7- Geometrical factor in heterogeneous ice nucleation

Here, we calculate the geometrical factor (f)[5] for MAGSS. The geometrical factor for a concave curvature is shown below:

$$f(m,x) = \frac{1}{2}[1-(\frac{1+mx}{g_c})^3 - x^3[2-3(\frac{x+m}{g_c})+(\frac{x+m}{g_c})^3] + 3mx^2(\frac{x+m}{g_c}-1)] \quad (14)$$

where

$$g_c = (1+2mx+x^2)^{\frac{1}{2}} \quad (15)$$

and m=cos(θ^{IW}), x=$\frac{R}{r_c}$, and $r_c = \frac{2\gamma_{IW}}{\Delta G_v}$. θ_{IW} denotes contact angle at the ice-water interface, R the radius of heterogenous nucleation site, γ_{IW} the interfacial energy at ice-water interface, ΔG_v the phase-change Gibbs energy. We plotted $f(m,x)$ as a function of x for different values of m in Fig. 19. Note that f=1 corresponds to homogeneous nucleation. At high values of x, $f(m,x)$ is independent of x.

Since the surface of MAGSS is smooth on the molecular scale, the value of R approaches infinity along with the value of x. To find the value of m for MAGSS, we considered two cases shown in Fig. 20. The first case considers a ferrofluid droplet on an ice substrate in a water medium. The second case considers an ice embryo on ferrofluid in a water medium. The interfacial force balance equations are written as:

$$\gamma^{IW} - \gamma^{IF} - \gamma^{FW} cos(\theta_1) = 0 \quad (16)$$

$$\gamma^{FW} - \gamma^{IF} - \gamma^{IW} cos(\theta_2) = 0 \quad (17)$$

where γ^{IF} the ice-ferrofluid interfacial tension, γ^{Fw} the ferrofluid-water interfacial tension. By subtraction of these two relations and knowing $\cos(\theta_2)$ is equal to m, one finds

$$m = (1 + cos\theta_1)\frac{\gamma^{FW}}{\gamma^{IW}} - 1 \tag{18}$$

We developed an experimental configuration similar to the one shown in Fig. 20(a) to measure θ_1. In these experiments, we measured the contact angle of ferrofluid on smooth ice in a water medium. This allows us to calculate the value of m. A glass beaker, thoroughly cleaned, was prepared. 10 mL of DI water was poured in the beaker and the beaker was placed on a cooling stage to form ice. The ice started to grow from the bottom of the beaker until the whole volume of water transformed into ice. The surface of the ice was completely smooth with an optical microscope. However, the ice surface may have some roughness at the nano-scale depending on the growth mechanism at the ice-water interface (Dendritic or flat solidification). Then, a ferrofluid droplet was deposited on the ice surface and the beaker was filled with water in a quasi-steady manner to avoid any further ice growth. The contact angle of the ferrofluid droplet on ice (θ_1) was measured with a digital camera (Canon DSLR) once the introduced water completely covered the ferrofluid droplet. Note that the temperature was measured at the ferrofluid-ice interface. As discussed surface tension of ferrofluid-water interface was measured with a tensiometer (Biolin Scientific, Sigma 701) equipped with a Du Nuoy ring. These surface tensions are tabulated in Table S1. The surface tension of the ice-water interface was extracted from [6] in which this interfacial tension is given as $\gamma^{IW}(T) = 23.24 \times (T/235.8)^{0.35}$ mNm^{-1}, where T is in Kelvin. With these measured values, we calculated value of m as a function of temperature. The calculation of m value allows us to theoretically predict the ice nucleation delay time and compare our experimental measurements

with the predicted values. The average ice nucleation delay time is written as

$$\tau_{av} = \frac{1}{J(T)} \tag{19}$$

where $J(T)$ denotes the rate of formation of critical ice embryo and is written as

$$J(T) = K(T) \times A \times exp(\frac{-\Delta G^*(T)}{k_b T}) \tag{20}$$

where $K(T)$ denotes the kinetic factor for diffusion of water molecules across the ice surface, A the water-solid contact area, $\Delta G^*(T)$ the Gibbs energy barrier of formation of critical ice embryo, and k_b the Boltzmann constant. The value of m plays a role in $\Delta G^*(T)$

$$\Delta G^* = \frac{8\pi\gamma_{IW}^3}{3\Delta G_v^2} f(m, x) \tag{21}$$

Once we measured the m value, we used the determined values of $K(T)$ (=2.0810^{29} $\times$ T $\times$ $exp(\frac{-892T}{(T-118)^2})$) and $A = \pi \times r^2$ as discussed in Eberle et al. [7] and determined τ_{av}. The surface tension of the ice-water interface play a significant role in these calculations. As discussed by Nemec [6], there is $\pm$ 1.1 mN/m uncertainty in this value. In Fig. 21, we compared our measurements with predictions. The measurements are within the uncertainty of the predictions. We should emphasize that in these calculations, we accurately considered the temperature-dependence of all parameters.

Supplementary References

1. Wilson, P. W. *et al.* Inhibition of ice nucleation by slippery liquid-infused porous surfaces (SLIPS). *Physical chemistry chemical physics : PCCP* **15**, 581–5 (2013).

2. Rosensweig, R. E. *Ferrohydrodynamics* (Dover Publications, Mineola, NY, 2014).

3. Timonen, J. V. I., Latikka, M., Leibler, L., Ras, R. H. a. & Ikkala, O. Switchable static and dynamic self-assembly of magnetic droplets on superhydrophobic surfaces. *Science (New York, N.Y.)* **341**, 253–7 (2013).

4. Wong, T.-S. *et al.* Bioinspired self-repairing slippery surfaces with pressure-stable omniphobicity. *Nature* **477**, 443–447 (2011).

5. Fletcher, N. H. Size Effect in Heterogeneous Nucleation. *The Journal of Chemical Physics* **29**, 572 (1958).

6. Nemec, T. Estimation of ice-water interfacial energy based on pressure-dependent formulation of classical nucleation theory. *Chemical Physics Letters* **583**, 64–68 (2013).

7. Eberle, P., Tiwari, M. K., Maitra, T. & Poulikakos, D. Rational nanostructuring of surfaces for extraordinary icephobicity. *Nanoscale* **6**, 4874–81 (2014).
